# Supplementary material for: Understanding genetic risk factors for common side effects of antidepressant medications
Source: Commun Med (Lond). 2021 Nov 9;1:45. doi: 10.1038/s43856-021-00046-8 (PMC9053224; doi:10.1038/s43856-021-00046-8)
Supplement: Supplementary file 1 — Description of Additional Supplementary Files [file 43856_2021_46_MOESM1_ESM.pdf]

Description of Additional Supplementary Files

File Name: Supplementary Data 1

Description: Prevalence of side effects across medications and demographic factors

File Name: Supplementary Data 2

Description: Fit statistics for structural equation models

File Name: Supplementary Data 3

Description: Association results between depression PRS and all side effects across medications

File Name: Supplementary Data 4

Description: Association results between BMI PRS and weight gain as a side effect across medications

File Name: Supplementary Data 5

Description: Association results between chronic headaches PRS and headaches as a side effect across medications

File Name: Supplementary Data 6

Description: Association results between Insomnia PRS and trouble sleeping as a side effect across medications
