# Supplementary material for: Understanding genetic risk factors for common side effects of antidepressant medications
Source: Commun Med (Lond). 2021 Nov 9;1:45. doi: 10.1038/s43856-021-00046-8 (PMC9053224; doi:10.1038/s43856-021-00046-8)
Supplement: Supplementary file 2 — Supplementary Information - Supplementary figures [file 43856_2021_46_MOESM2_ESM.pdf]

Supplementary Figures for "Understanding genetic risk factors for common side effects of antidepressant medications"

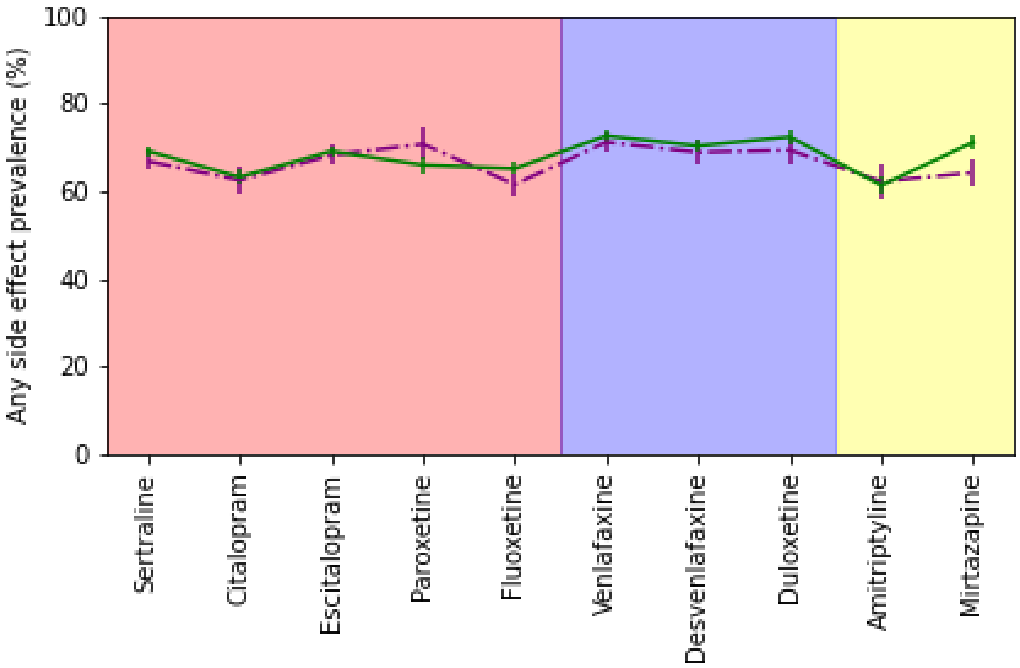

Supplementary Figure 1 Prevalence of any side effect across medications

Plot depicting the prevalence and 95% confidence interval for any side effects across the ten medications under study.

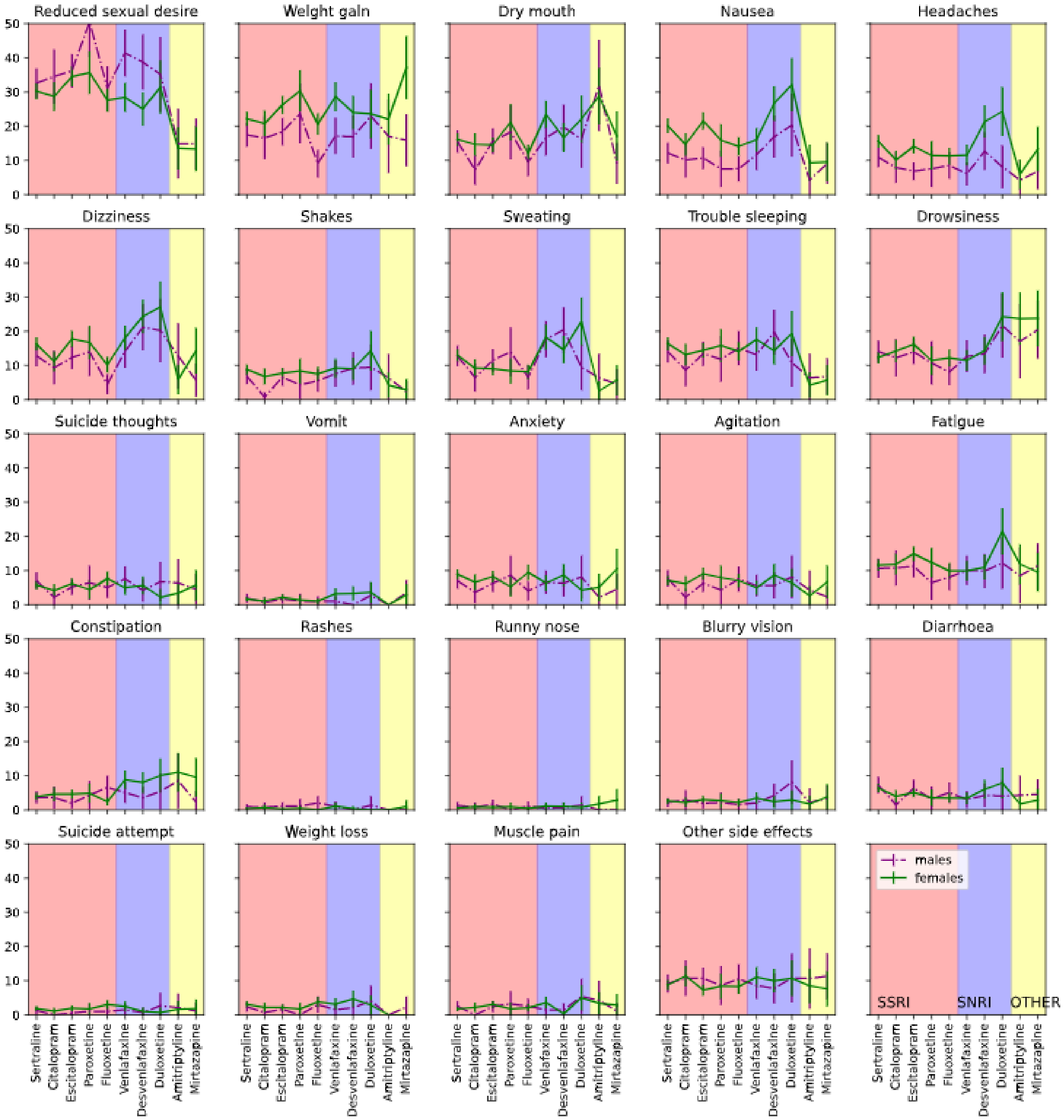

Supplementary Figure 2. Side effect prevalence sensitivity analysis

Side effect prevalence were estimated using only a subsample of participants who only reported taking one antidepressants. Thus, the effects would be less confounded from different course of illness and from combinations of antidepressants.

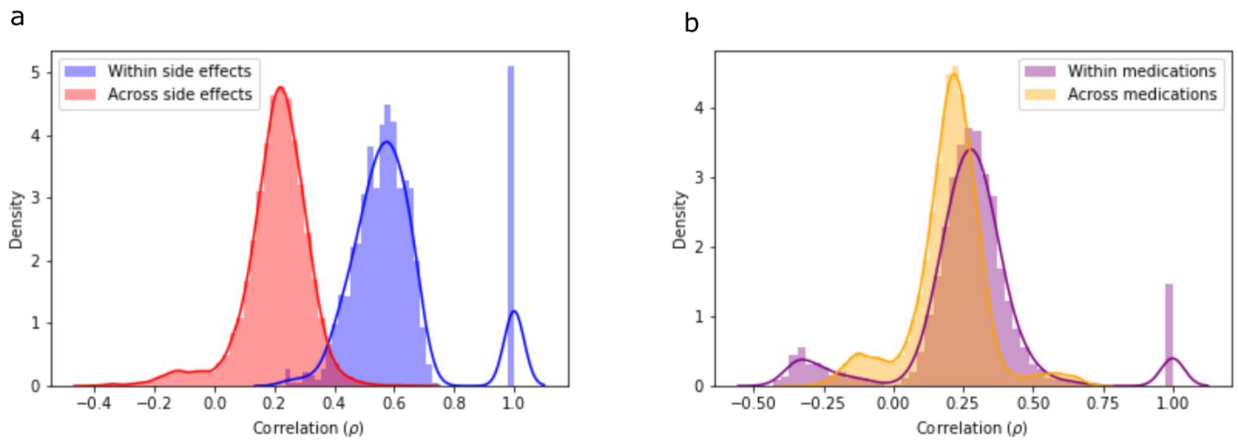

Supplementary Figure 3. Side effect correlation distribution

Density plots depicting the kernel density estimates based on the distribution of side effects correlations shown in Figure 2. a) Data split between the correlations for the same side effects across medications (blue) or different side effects within and across medications red. b) Data split between the correlations for different side effects within the same medication, or across medications.

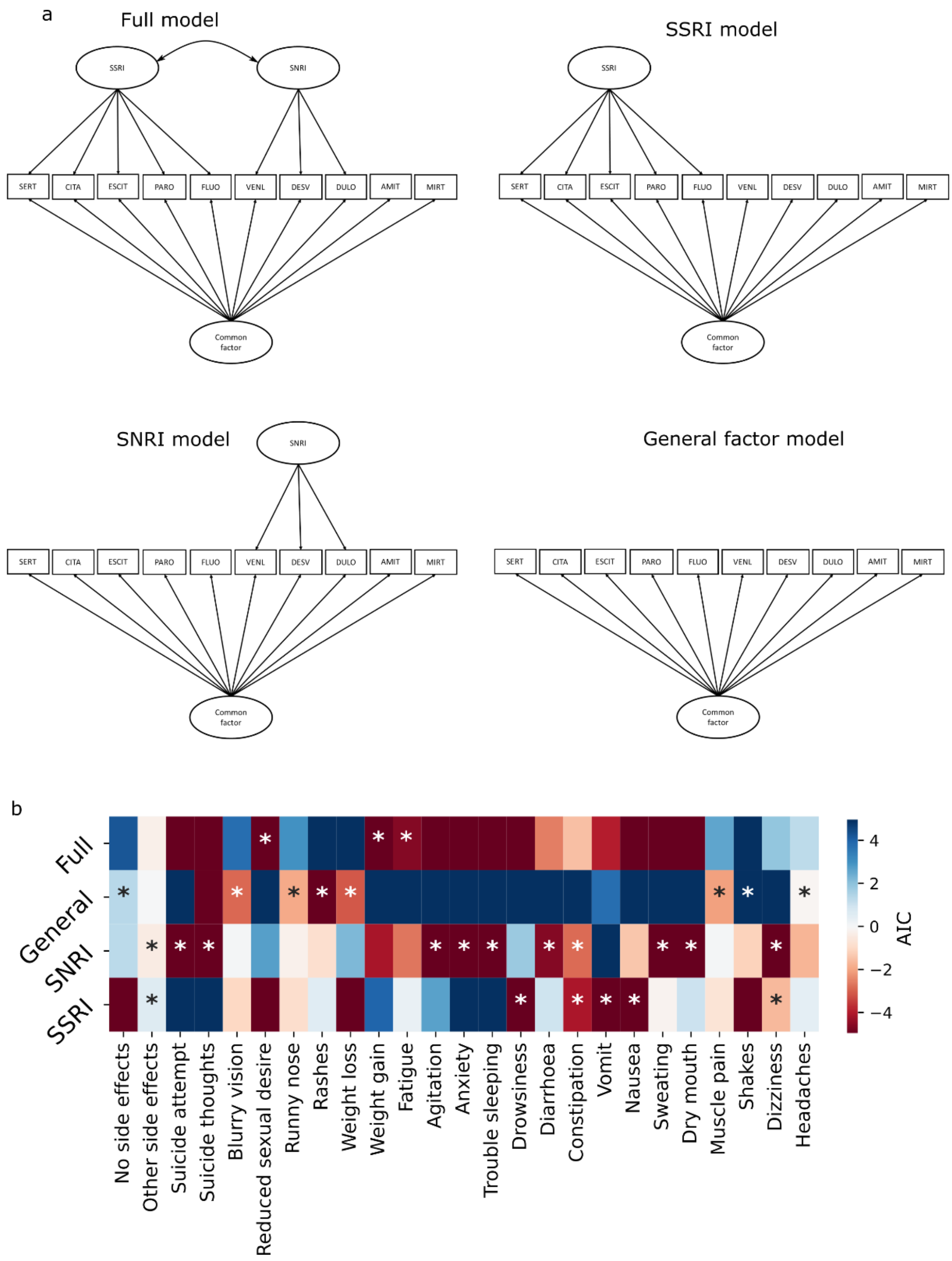

Supplementary Figure 4. SEM analysis results

a) Path diagrams of the structural equation models fit via full information maximum likelihood using OpenMx. The manifest variables represent a reported side effect for each medication. b) Heatmap comparing the models Akaike information criterion (AIC). AIC values were mean-centred to display them on a comparable scale; lower values represent better model fit. Stars mark the reduced model which would be chosen over the full model given their likelihood ratio test p-value. When two models are to be chosen, the most parsimonious one (if any) is preferred. **Supplementary Table 2** contains the relevant AIC values and likelihood ratio test p-values as well as the preferred model by each approach.

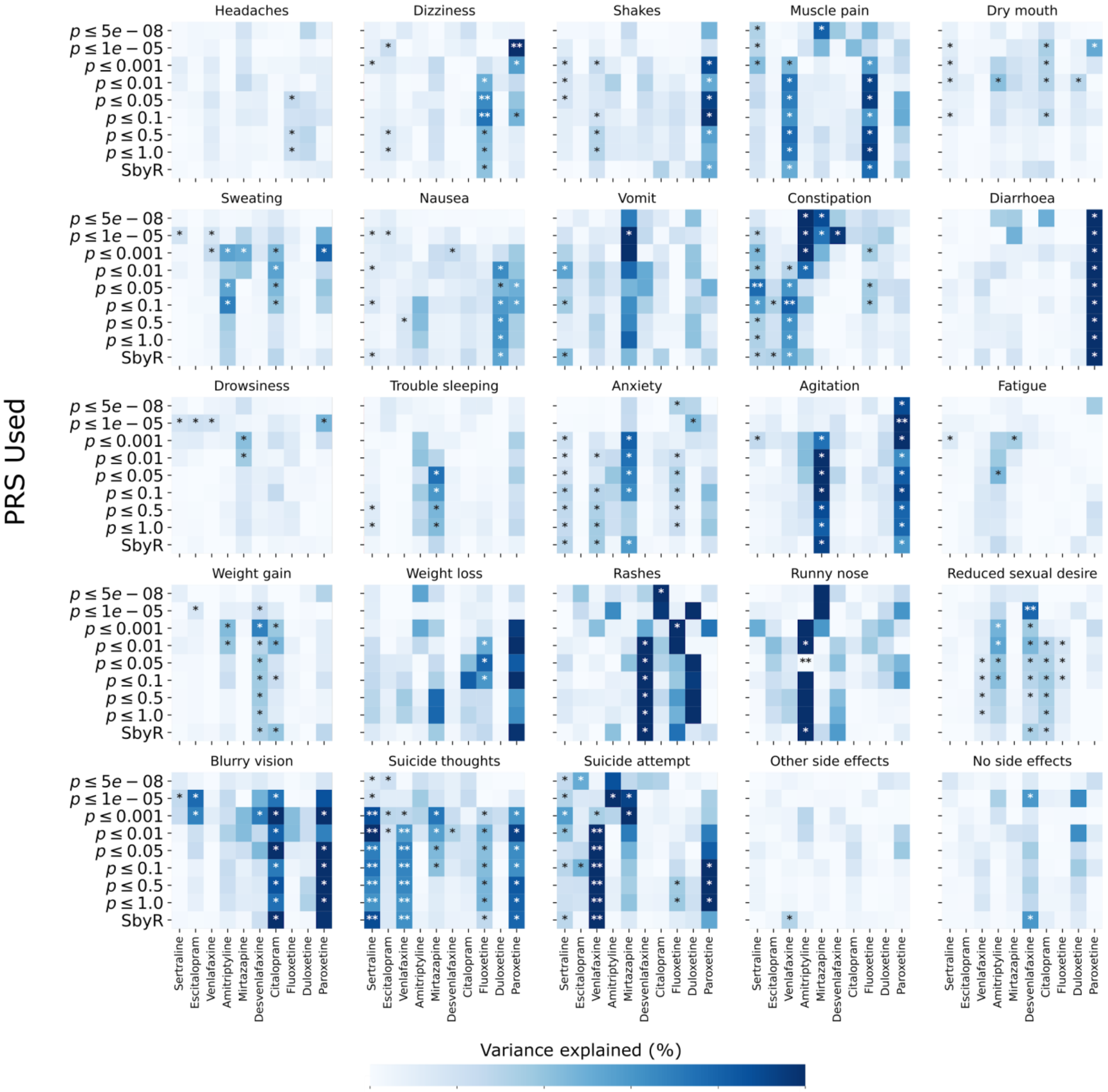

Supplementary Figure 5. Depression PRS prediction of side effects

Heatmaps showing the results of depression PRS predicting side effects across antidepressant medication. Labels of the y axis show the p-value inclusion threshold for PRS calculation when using clumping and thresholding or *SbyR* when showing results for SBayesR PRS.

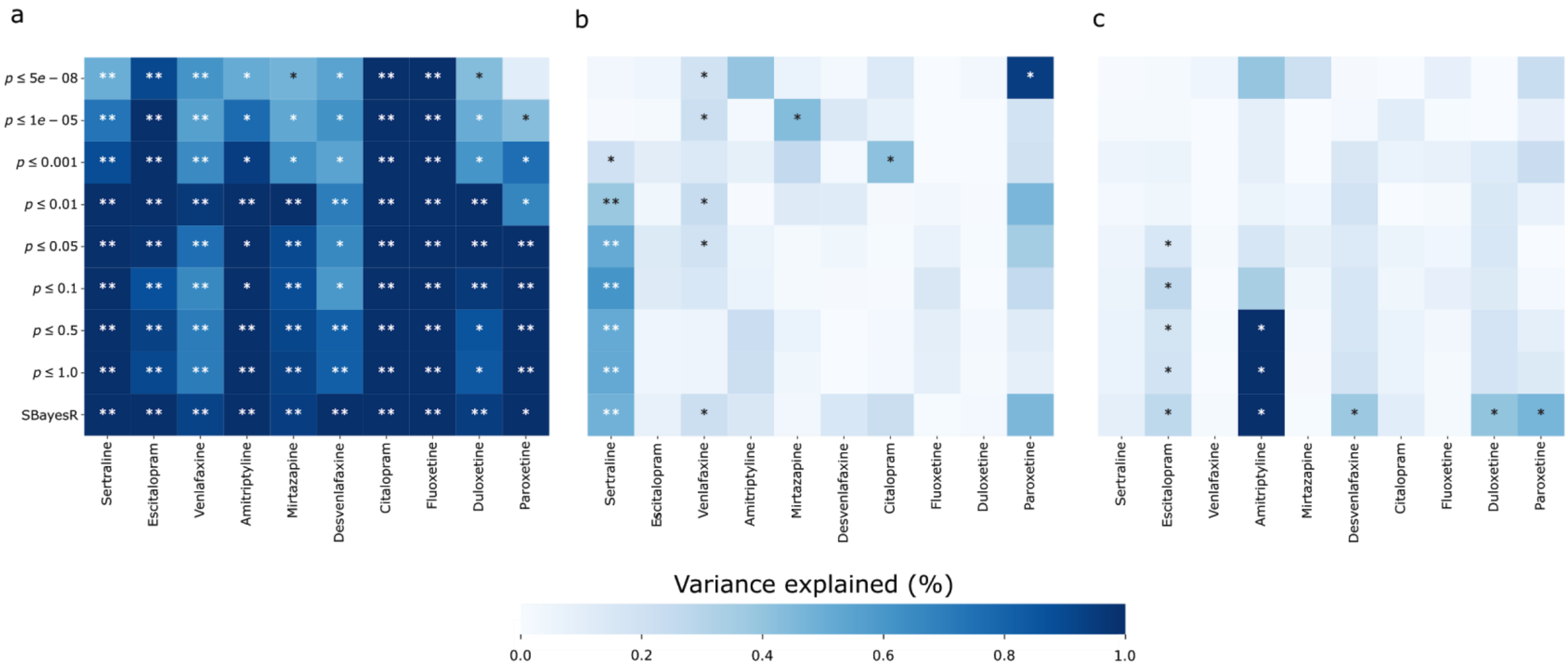

Supplementary Figure 6. Genetic factors underlying headaches, insomnia and BMI

Heatmaps showing the results (variance explained) of BMI PRS predicting weight gain (a); headaches PRS predicting headaches (b); and insomnia PRS predicting insomnia (c) as side effects from antidepressants.

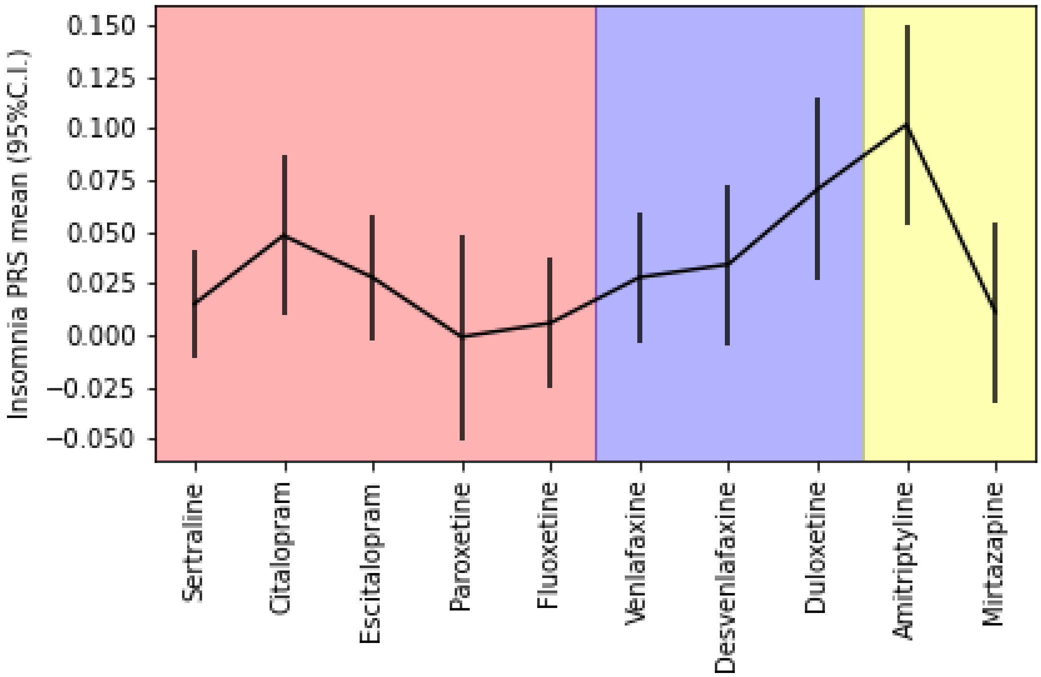

Supplementary Figure 7. Insomnia PRS distribution across medications

Plot depicting the mean and 95% confidence interval for insomnia PRS across participants reporting taking the different medications under study. Note the higher insomnia PRS for participants taken amitriptyline; a pattern expected given its use to treat insomnia.
