# Supplementary material for: Understanding genetic risk factors for common side effects of antidepressant medications
Source: Commun Med (Lond). 2021 Nov 9;1:45. doi: 10.1038/s43856-021-00046-8 (PMC9053224; doi:10.1038/s43856-021-00046-8)
Supplement: Supplementary file 10 — Reporting Summary [file 43856_2021_46_MOESM10_ESM.pdf]

nature research

Corresponding author(s): Adrian Campos and Miguel Renteria

Last updated by author(s): Sep 28, 2021

Reporting Summary

Nature Research wishes to improve the reproducibility of the work that we publish. This form provides structure for consistency and transparency in reporting. For further information on Nature Research policies, see our Editorial Policies and the Editorial Policy Checklist.

Statistics

For all statistical analyses, confirm that the following items are present in the figure legend, table legend, main text, or Methods section.

n/a	Confirmed	
☐	☒	The exact sample size (n) for each experimental group/condition, given as a discrete number and unit of measurement
☐	☒	A statement on whether measurements were taken from distinct samples or whether the same sample was measured repeatedly
☐	☒	The statistical test(s) used AND whether they are one- or two-sided<br>*Only common tests should be described solely by name; describe more complex techniques in the Methods section.*
☐	☒	A description of all covariates tested
☐	☒	A description of any assumptions or corrections, such as tests of normality and adjustment for multiple comparisons
☐	☒	A full description of the statistical parameters including central tendency (e.g. means) or other basic estimates (e.g. regression coefficient) AND variation (e.g. standard deviation) or associated estimates of uncertainty (e.g. confidence intervals)
☐	☒	For null hypothesis testing, the test statistic (e.g. F, t, r) with confidence intervals, effect sizes, degrees of freedom and P value noted<br>*Give P values as exact values whenever suitable.*
☒	☐	For Bayesian analysis, information on the choice of priors and Markov chain Monte Carlo settings
☒	☐	For hierarchical and complex designs, identification of the appropriate level for tests and full reporting of outcomes
☐	☒	Estimates of effect sizes (e.g. Cohen's d, Pearson's r), indicating how they were calculated

Our web collection on statistics for biologists contains articles on many of the points above.

Software and code

Policy information about availability of computer code

Data collection	Data collection was performed using qualtrics.
Data analysis	R v3.6.1 inclduing the libraries lavaan and psych<br>Python 3 modules seaborn, matplotlib<br>PLINK v1.96b for polygenic risk scoring<br>GCTB v2.03beta for SBayesR analysis<br>This study used python (3.6) and R (3.6.2) code for data analysis. Code is available as HTML notebooks or R scripts on-line at doi: 10.5281/zenodo.5533372.

For manuscripts utilizing custom algorithms or software that are central to the research but not yet described in published literature, software must be made available to editors and reviewers. We strongly encourage code deposition in a community repository (e.g. GitHub). See the Nature Research guidelines for submitting code & software for further information.

Data

Policy information about availability of data

All manuscripts must include a data availability statement. This statement should provide the following information, where applicable:

- Accession codes, unique identifiers, or web links for publicly available datasets
- A list of figures that have associated raw data
- A description of any restrictions on data availability

Summary data on prevalence and effects described in this manuscript is available in the supplementary data. Source data for the main figures of the manuscript is available in supplementary data 1-6 or online doi: 10.5281/zenodo.5533372. GWAS summary statistics used in this study are publicly available. For MDD the data were obtained from the PGC leaving out the QIMR cohorts. The full results are available at https://www.med.unc.edu/pgc/download-results/. The insomnia

summary statistics are available at https://ctg.cncr.nl/software/summary_statistics. The BMI summary statistics are available at https://portals.broadinstitute.org/collaboration/giant/index.php/GIANT_consortium_data_files. The chronic headaches summary statistics are available online at doi: 10.5281/zenodo.5533372. Furthermore, all of the effect sizes for PGS are available online at the PGS Catalogue under publication ID PGP000238. Access to the AGDS data is restricted due to the ethical guidelines governing the study, but may be accessible following ethical review and data transfer agreements, please contact Nicholas Martin (nick.martin@qimrberghofer.edu.au) with any queries related to accessing AGDS data.

Field-specific reporting

Please select the one below that is the best fit for your research. If you are not sure, read the appropriate sections before making your selection.

☒ Life sciences ☐ Behavioural & social sciences ☐ Ecological, evolutionary & environmental sciences

For a reference copy of the document with all sections, see nature.com/documents/nr-reporting-summary-flat.pdf

Life sciences study design

All studies must disclose on these points even when the disclosure is negative.

Sample size	No a priori sample size calculation was performed. Recruitment was planned as to maximize sample size.
Data exclusions	Participants were excluded if they did not have a diagnosis of depression. Complete case analysis was performed for some sections (as detailed in the manuscript), thus participants with missing data were excluded accordingly.
Replication	Our findings need to be replicated on other samples with data on antidepressant exposures, adverse side-effects and genotype or whole genome sequencing. We are not aware of an independent sample with such characteristics readily available.
Randomization	No randomization was performed because this is not an interventional study
Blinding	Blinding is not relevant as this was a retrospective study (not an experimental one).

Reporting for specific materials, systems and methods

We require information from authors about some types of materials, experimental systems and methods used in many studies. Here, indicate whether each material, system or method listed is relevant to your study. If you are not sure if a list item applies to your research, read the appropriate section before selecting a response.

Materials & experimental systems

n/a	Involved in the study	
☒	☐	Antibodies
☒	☐	Eukaryotic cell lines
☒	☐	Palaeontology and archaeology
☒	☐	Animals and other organisms
☐	☒	Human research participants
☒	☐	Clinical data
☒	☐	Dual use research of concern

Methods

n/a	Involved in the study	
☒	☐	ChIP-seq
☒	☐	Flow cytometry
☒	☐	MRI-based neuroimaging

Human research participants

Policy information about studies involving human research participants

Population characteristics	The sample comprises Australian adults that have been treated for depression (N=20,941). The majority of participants were female (15,830 ~75%) which were also younger (mean age 41.41) than males (mean age 47.99)
Recruitment	Briefly, 14.3% of volunteers were recruited by mail invitations distributed by the Australian Department of Human Services (DHS) and encouraging individuals who had previously used prescription antidepressants in the last 4.5 years to participate. Secondly, a nationwide media publicity campaign was broadcast which targeted individuals who are currently or have sought medical attention by a psychiatrist or a psychologist for clinical depression. Recruited participants were directed to the study website to complete consent forms before answering the instruments. Once the questions had been completed, a GeneFix GFX-02 DNA extraction kit (Isohelix plc) was sent to participants to collect 2mL of saliva for DNA extraction.
Ethics oversight	The protocol for approaching participants through the DHS, enrolling them in the study and consenting for all phases of the study (including invitation to future related studies) and accessing MBS and PBS records was approved by the Ethics Department of the Department of Human Services. The QIMR Human Research Ethics Committee also approved all protocols under project number 2118. The study presented here falls within the scope of the analyses reviewed and approved under project 2118.

Note that full information on the approval of the study protocol must also be provided in the manuscript.
